# Quantitative MRI Findings and Their Relationship to Muscle Histopathology and Ambulatory Clinical Function in Duchenne Muscular Dystrophy

**DOI:** 10.1002/jcsm.70205

**Published:** 2026-01-25

**Authors:** Yanyu Lu, Liang Yin, Chang Liu, Qingyue Yuan, Zhihao Xie, Jianan Liao, Siwei Chen, Qiang Gang, Yawen Zhao, Lingchao Meng, Wei Zhang, Jiangxi Xiao, Zhaoxia Wang, Yun Yuan, Zhiying Xie

**Affiliations:** ^1^ Department of Neurology Peking University First Hospital Beijing China; ^2^ Department of Interventional Radiology the First Affiliated Hospital of University of Science and Technology of China Hefei China; ^3^ Department of Epidemiology and Health Statistics, School of Public Health Yangzhou University Yangzhou China; ^4^ Department of Radiology Peking University First Hospital Beijing China

**Keywords:** Duchenne muscular dystrophy, fatty infiltration, histopathology, IDEAL‐IQ, quantitative magnetic resonance imaging

## Abstract

**Background:**

Iterative decomposition of water and fat with echo asymmetry and least‐squares estimation quantitation (IDEAL‐IQ), a quantitative 6‐point Dixon magnetic resonance imaging (MRI) sequence, has been increasingly used for quantifying muscle fat fraction (FF) in neuromuscular disorders. However, its utility for correlating FF with disease severity and mapping spatiotemporal disease progression in Duchenne muscular dystrophy (DMD) requires further investigation.

**Methods:**

In 10 patients with dystrophinopathies (seven DMD and three BMD) we correlated the muscle FF with the histopathological fatty infiltration. IDEAL‐IQ MRI images of 19 individual lower limb muscles were acquired from 133 DMD patients and 41 healthy controls. Ambulatory function tests, including three timed function tests (TFTs) and North Star Ambulatory Assessment (NSAA), were performed. We investigated the spatial distribution of fatty infiltration across five axial MRI slices. Disease progression patterns were analysed using a piecewise linear model and a normal cumulative distribution function model.

**Results:**

A significant positive correlation between muscle FF on MRI and histopathologic fatty infiltration was observed (ρ = 0.98, *p* < 0.001). A mild to moderate correlation was noted for the FF of 19 individual muscles, excluding the sartorius and gracilis muscles, with TFTs (ρ = 0.27–0.60, *p* < 0.001) and NSAA (ρ = −0.32–−0.73, *p* < 0.001). All the eight individual muscles, except the tibialis posterior muscle, showed an inhomogeneous fatty infiltration pattern with higher FF in muscle ends compared to bellies. Thigh muscle FF aligned well with both piecewise linear and sigmoidal models, showing non‐linear increases with disease duration. The piecewise linear model identified an average inflection point for thigh muscle FF at 7.3 (range, 5.2–7.6) years, with average annual FF increase of 1.7% before and 6.7% after the point in DMD. The average μ of DMD patients for thigh muscles was 11.4 years (±2.8), occurring 7.6 years earlier compared to BMD patients.

**Conclusions:**

Our study demonstrates a significant relationship between muscle FF on IDEAL‐IQ MRI and histopathologic muscle fatty infiltration, as well as ambulatory clinical function in DMD. DMD patients exhibit a distinct and inhomogeneous fat infiltration pattern of lower limb muscles.

## 1 Introduction

Duchenne muscular dystrophy (DMD) is the most common muscular dystrophy [1], resulting from loss‐of‐function variants in the *dystrophin* gene. Like most muscular dystrophies, the pathology of DMD is characterized by repeated cycles of myofibre necrosis and regeneration, accompanied by inflammation, eventually leading to the replacement of myofibres with fat and connective tissue [2]. Although currently no cure exists, several promising therapies are under development [3]. To evaluate the efficacy of the proposed therapies, there remains a compelling need for non‐invasive and reliable assessment tools that are sensitive to histopathologic changes of muscular dystrophies. Chemical shift‐encoded magnetic resonance imaging (CSE‐MRI), including Dixon‐based techniques, is widely used to quantify fat fraction (FF) and track disease progression in neuromuscular disorders [4]. These methods leverage the difference in resonance frequencies between water and fat protons to generate separate water/fat images, enabling precise FF quantification [5]. Modern multi‐echo Dixon sequences (e.g., 6‐point Dixon) further improve accuracy by correcting for confounding factors such as T2 decay and field inhomogeneity [6, 7]. This improves accuracy in evaluating heterogeneous muscles, especially in paediatric populations where motion and susceptibility artefacts are common [8].

CSE‐MRI techniques have been validated on various platforms, such as Philips Healthcare mDIXON Quant, Siemens Healthcare Dixon‐VIBE, GE Healthcare iterative decomposition of water and fat with echo asymmetry and least‐squares estimation quantitation (IDEAL‐IQ) and demonstrate strong correlation with histopathologic fat replacement in muscular dystrophies [9, 10], supporting their utility as objective biomarkers. Despite this, given the muscle‐specific involvement across various muscular dystrophies and potential variations in MRI devices or magnetic fields, the relationship between IDEAL‐IQ MRI‐derived FF and histopathologic fatty infiltration in DMD requires further investigation. Moreover, spatial heterogeneity in fat replacement—observed in DMD [11], limb‐girdle muscular dystrophy 12 [12] and other dystrophies [13, 14], together with findings from non‐linear models of disease progression in DMD [15, 16], underscores the importance of multimodal, multidimensional and muscle‐specific involvement analyses of MRI data to enhance the overall understanding of disease‐specific patterns and progression of muscle involvement.

This study correlates muscle FF on IDEAL‐IQ MRI with histopathologic fatty infiltration in the vastus lateralis muscle of dystrophinopathy patients to validate the reliability and effectiveness. We then examined the relationship between muscle MRI FF and clinical assessments in a large cohort of DMD patients. Furthermore, we present a comprehensive analysis of the spatial distribution and temporal progression of lower limb muscle involvement in DMD, aiming to demonstrate the utility of IDEAL‐IQ in capturing these disease characteristics. To better characterize the heterogeneity of disease progression, a subset of patients with Becker muscular dystrophy (BMD) was also included in the temporal modelling.

## 2 Methods

### 2.1 Patients and Study Design

A total of 133 patients with DMD, 31 patients with BMD and 41 male controls were recruited at Peking University First Hospital Neuromuscular Center (Figure S1). To validate the reliability and effectiveness of IDEAL‐IQ MRI for detecting histopathologic muscle fatty infiltration, 10 patients with dystrophinopathy (7 DMD and 3 BMD) underwent muscle biopsies. The inclusion criteria for DMD patients were as follows: (1) clinical presentation of proximal muscle weakness evident by age 5 and (2) harbouring frameshift or nonsense *DMD* variants and/or complete or near‐complete deficiency of dystrophin, especially the dystrophin‐C (carboxyl‐terminal). The clinical criteria for DMD and BMD were defined according to the European Neuromuscular Centre guidelines [17]. Patients on glucocorticoids or with MRI contraindications were excluded. All DMD participants underwent IDEAL‐IQ MRI of the pelvis, thigh and leg muscles, followed by clinical function assessments within 1 week.

This study was approved by the Ethics Committee at Peking University First Hospital (2023 109‐002). Informed consent was obtained from adult participants or the parents/guardians of minors.

### 2.2 MRI Data Acquisition and Analysis

All MRI data were acquired using a 3 T system (GE Discovery 750) with a 32‐channel phased‐array torso coil. Participants lay in a feet‐first supine position. The scanning protocol included axial T1‐weighted spin echo sequences (repetition time [TR] 590–740 ms, echo time [TE] 9–10 ms, echo train length 2, slice thickness 5 mm, interslice gap 1 mm, field of view [FOV] 28 × 42 cm^2^, matrix 512 × 512, acquisition time 3 min) and IDEAL‐IQ sequences (TR 6.9 ms, TE 3.2 ms, echo train length 6, slice thickness 6 mm, FOV 30 × 30 cm^2^, matrix 160 × 160, flip angle 3°, number of excitations 2, bandwidth 111.11 KHz, acquisition time 1 min 40 s). For IDEAL‐IQ, 50 sections were acquired using these parameters for the pelvis and thigh (from the iliac crest to femoral condyles) and the leg (from tibial plateaus to tibiotalar joints). The total duration of the MRI examination was approximately 30 min. The imaging data were transferred to an imaging workstation (Advantage Windows 4.6). IDEAL‐IQ Fat Fraction images were selected to outline the regions of interest (ROIs), and muscle FF values were automatically calculated.

In the 10 patients who underwent IDEAL‐IQ MRI followed by muscle biopsies, the chosen MRI sections were marked using the system's infrared positioning light following the methodology outlined in previous research [18]. The biopsy position was marked on the patient's skin with a pen (approximately 5 mm diameter). On the imaging workstation, circular ROIs (8 mm diameter) were drawn to encompass the biopsy site. To evaluate the consistency and reproducibility of MRI measurements, ROIs were independently drawn by three experienced observers (Drs Zhaoxia Wang, Yun Yuan and Zhiying Xie), who were blinded to patient information [19]. They repeated the measurements in a randomized order after 2 weeks. For DMD patients and healthy controls, a radiologist (Dr Liang Yin) manually drew ROIs on FF maps of 19 muscles on the right side of the lower limb. Four section levels were chosen for ROI placement, containing the largest area of visible muscles with good differentiation of the different muscle compartments (Table S1 and Figure S2).

In 31 randomly selected patients with DMD and 24 healthy controls, we analysed muscle fatty infiltration along the full length of eight muscles: rectus femoris, vastus lateralis, biceps femoris long head, tibialis anterior, extensor digitorum longus, peroneal group, tibialis posterior and soleus. For each muscle, five axial MRI images were selected based on thigh and leg lengths. The slice with the maximal cross‐sectional area (maxCSA) of the selected muscle was identified and fixed as the central slice. Two proximal and two distal slices were then chosen by dividing the proximal and distal muscle sections into three equal lengths, respectively, based on the overall muscle length.

### 2.3 Muscle Biopsies

Open‐muscle biopsies were obtained from the vastus lateralis muscle at previously marked locations. Each patient underwent a single biopsy under local anaesthesia with a 2 cm incision parallel to the muscle belly. After separating the skin, subcutaneous fat and deep fascia, a muscle tissue sample measuring approximately 0.5 × 0.5 × 1 cm^3^ was excised. The biopsies were immediately frozen in isopentane cooled in liquid nitrogen and then stored at −80°C. Serial cross‐sections, 6 μm thick, were cut using a microtome cryostat. Histological, histochemical and immunohistochemical staining were performed [20]. Three representative haematoxylin and eosin staining images of each biopsy were captured at 200× magnification. We then used ImageJ software to manually delineate fat tissue and calculate the percentage of fatty infiltration. The average percentage from the three images represented the histopathologic fatty infiltration of each biopsied muscle.

### 2.4 Clinical Functional Assessments

TFTs and NSAA were used for clinical functional assessments. TFTs included the 10 m walk/run, 4‐stair climb and supine‐to‐stand tests, which are reproducible and measure functional capability in ambulatory patients. Patients were encouraged to complete tasks as quickly as possible, with an average of three trials used for analysis. The maximum allowable time was 30 s for each test [21]. The NSAA, a validated unidimensional functional scale for ambulant DMD boys, measures motor abilities across 17 items, with scores ranging from 0 (*non‐ambulant*) to 34 (*fully independent*) [22].

### 2.5 Statistical Analysis

Data were analysed using GraphPad Prism 8 and RStudio (Version 4.3.1). Demographic data, including age, age at onset, disease duration, Medical Research Council scale percentage (MRC%), serum creatine kinase levels, muscle MRI FF values and clinical functional assessments, were reported as medians with interquartile ranges (IQRs). The Shapiro–Wilk test assessed data distribution normality. An independent *t*‐test was conducted to compare age differences, whereas the Mann–Whitney test was used to compare the FF differences between DMD patients and healthy controls.

The correlation between muscle MRI FF and histopathologic fatty infiltration percentages was visualized by scatterplots and quantified using Spearman's correlation coefficients, Bland–Altman plots and linear regression analysis. The reproducibility and consistency of ROI measurements were assessed using the intraclass correlation coefficient (ICC) among three observers. The relationship between muscle MRI FF and clinical functional assessments was assessed using Spearman's correlation and linear regression analysis. The Wilcoxon signed‐rank test or Friedman test was performed to compare muscle MRI FF values across various muscle regions (proximal, mild‐proximal, central, mild‐distal and distal). *p* < 0.05 was considered statistically significant.

To investigate patterns of FF change with age, three‐knot b‐splines were initially used. A piecewise linear (PL) model was then constructed for MRI data from patients with DMD and BMD as follows:

(1) $$\begin{array}{c} \mathrm{FF}=k_{1}*x+y_{0}-k_{1}*x_{0},x\leq x_{0}\\ \mathrm{FF}=k_{2}*x+y_{0}-k_{2}*x_{0},x\geq x_{0} \end{array}$$

In Equation (1), k_1_ and k_2_ represent the slopes of the two segments of the function, x_0_ represents the cut‐off age, and y_0_ represents the FF value at age x_0_.

Following previous research [23], a normal cumulative distribution function (NCDF) was also used to model MRI FF progression:

(2) $$E\left[Y\left(t\right)\right]=A*\int \frac{\exp\left\{-\frac{{\left(t-\mu \right)}^{2}}{2\sigma^{2}}\right\}\mathrm{dt}}{\sigma \sqrt{2\pi }}+C$$

In Equation (2), the FF value at age t is estimated by E[Y(t)], where μ represents the mid‐value of t (age) through FF progression, which is normally located in the maximal gradient point of the function (greatest progression rate constant and half‐maximum of MRI FF), σ is the time constant, A is the FF total amplitude and C is the FF initial value. MRI FF measures from 12 thigh muscles and tibialis anterior muscle were modelled using Equation (2).

We initially implemented two variants of the NCDF model differing in the number of free parameters. NCDF1 represents a four‐parameter model, in which A, μ, σ and C are all estimated during model fitting. NCDF2 is a simplified two‐parameter model that retains only μ and σ as free parameters, whereas A is fixed as the maximum value observed in the corresponding muscle dataset, and C is set to 0.01. To compare these models with each other and with the PL model, we used the Akaike Information Criterion (AIC), where a lower AIC value indicates a better model fit. Because NCDF1 and NCDF2 are nested models, we applied the likelihood ratio test to assess their relative performance, with *p* < 0.05 considered statistically significant. NCDF2 and the PL models are independent and were compared directly using the difference in AIC values (a difference of > 10 between models is considered significant).

## 3 Results

### 3.1 Correlation Between IDEAL‐IQ MRI and Histopathologic Findings

**FIGURE 1:**
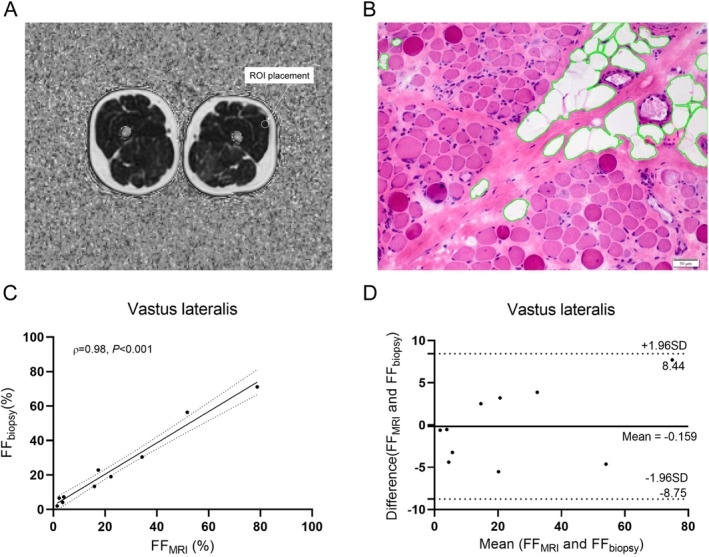
The correlation between muscle FF on IDEAL‐IQ MRI and histopathologic muscle fatty infiltration. (A) Axial IDEAL‐IQ MR images showed the vastus lateralis muscle and the ROI placement. (B) Transverse section of the vastus lateralis muscle biopsy stained with haematoxylin–eosin revealed increased variability in fibre size, fibrosis and loss of muscle tissue with fatty replacement. The fatty infiltration percentage calculated at histopathology was 6.9%. (C) Scatterplot and regression line exhibited a robust correlation (ρ = 0.98, *p* < 0.001) between histopathologic fatty infiltration percentage and MRI FF. Solid line represents the regression line, whereas dashed lines denote the 95% confidence interval. (D) Bland–Altman plot depicting the consistency of fatty infiltration estimated at histopathology and FF at IDEAL‐IQ MR imaging. All data points fall within the limits of agreement (dotted lines), corresponding to ±1.96 standard deviations from the mean. FF, fat fraction; ROI, region of interest.

Of the 10 patients with dystrophinopathies (median age, 5.95 years), seven were DMD, and three were BMD. IDEAL‐IQ MRI revealed a median FF of 16.63% (IQR 3.23%–38.68%), which was comparable to histopathology findings (median 16.19%, IQR 6.00%–36.93%) (Table S2). We observed a strong correlation between muscle MRI FF and histopathologic fatty infiltration (ρ = 0.98, *p* < 0.001), with good agreement (slope = 0.91) (Figure 1). The Bland–Altman plot showed that all points were within the ±1.96 standard deviations (range −8.76%–8.44%). Interobserver and intraobserver reproducibility for FF values was excellent (ICCs > 0.75) (Tables S3 and S4).

### 3.2 Clinical Characteristics of the 133 DMD Patients

The clinical features of the 133 DMD patients were summarized in Table 1. The median age was 8.3 years (IQR 6.7–9.5 years), onset age 3.5 years (IQR 3.0–4.5 years) and disease duration 4.7 years (IQR 3.0–5.8 years). Onset symptoms were associated with lower limb weakness in all patients, including frequent falls, abnormal gait and difficulties in running, jumping, climbing stairs and rising from the floor. Sixty‐four patients also experienced activity‐induced fatigue and/or myalgia. Physical examination revealed proximal muscle weakness in 124 patients, with 90 also showing distal muscle weakness. The median MRC% was 88.6% (IQR 77.1%–94.2%). Serum creatine kinase levels were markedly elevated (median 11526.5 IU/L, IQR 8000–18 270 IU/L). The median NSAA scores were 19 (IQR 11–26). Some patients were unable to complete certain TFTs within the allotted time: 13 patients for the 10 m walk/run, 19 for the 4‐stair climb and 30 for the supine‐to‐stand. The median times were 5.1 s (IQR 4.4–6.6 s) for the 10 m walk/run, 3.9 s (IQR 2.0–6.3 s) for the 4‐stair climb and 5.9 s (IQR 3.9–9.1 s) for the supine‐to‐stand. Patients who were unable to complete all three TFTs and scored 0 on the NSAA were defined as having lost ambulation (LoA), resulting in a total of 13 non‐ambulatory patients.

**TABLE 1:** Clinical characteristics, functional assessments and FF values of DMD patients and controls.

Muscle	DMDs (*n* = 133)	Controls (*n* = 41)	*p*
Age (years)	8.3 (6.7–9.5)	9.0 (7–10.6)	0.09
Age onset (years)	3.5 (3.0–4.5)	—	
Disease duration (years)	4.7 (3.0–5.8)	—	
MRC%	88.6 (77.1–94.2)	—	
Creatine kinase (IU/L)	11526.5 (8000–18 270)	—	
Clinical functional assessments		—	
NSAA	19 (11–26)	—	
10‐m walk/run (s)	5.1 (4.4–6.6)	—	
4‐stair climb (s)	3.9 (2.0–6.3)	—	
Supine‐to‐stand (s)	5.9 (3.9–9.1)	—	
Non‐ambulant, *n* (%)	13 (9.8%)	—	
FF (%)			
Gluteus maximus	32.3 (20.1–48.9)	5.2 (3.8–6.8)	**< 0.001**
Rectus femoris	9.9 (6.1–26.1)	1.9 (1.4–2.8)	**< 0.001**
Vastus intermedius	9.4 (3.8–26.7)	1.6 (1.2–2.1)	**< 0.001**
Vastus lateralis	15.0 (6.7–40.0)	2.1 (1.5–2.6)	**< 0.001**
Vastus medialis	16.4 (6.2–33.2)	1.9 (1.6–3.2)	**< 0.001**
Sartorius	5.7 (3.7–9.7)	4.3 (3.1–5.8)	**< 0.001**
Gracilis	3.9 (2.3–5.5)	3.2 (2.1–4.7)	**< 0.001**
Adductor longus	2.5 (1.3–6.7)	1.6 (1.2–1.9)	**< 0.001**
Adductor magnus	22.4 (10.9–49.0)	2.2 (1.6–3.1)	**< 0.001**
Semimembranosus	6.0 (3.8–13.3)	2.4 (1.8–3.4)	**< 0.001**
Semitendinosus	7.8 (4.9–14.6)	2.5 (1.4–3.3)	**< 0.001**
Biceps femoris long head	10.3 (5.4–21.8)	2.5 (2.0–3.8)	**< 0.001**
Tibialis anterior	3.0 (1.6–5.2)	1.6 (1.3–1.9)	**< 0.001**
Extensor digitorum longus	3.2 (2.2–6)	2 (1.6–2.6)	**< 0.001**
Peroneal group	6.1 (3.6–14.7)	2.5 (1.8–3.4)	**< 0.001**
Tibialis posterior	2.1 (1.3–3.6)	2.0 (1.2–2.5)	0.11
Soleus	4.7 (3.2–8.8)	2.3 (1.8–2.8)	**< 0.001**
Medial head of gastrocnemius	4.8 (3.2–11.2)	2.1 (1.5–2.7)	**< 0.001**
Lateral head of gastrocnemius	6.7 (3.6–18.9)	2.3 (1.8–2.8)	**< 0.001**

Abbreviations: FF, fat fraction; MRC%, the proportion of Medical Research Council scale = total MRC/[5 × 7] × 100%; NSAA, North Star Ambulatory Assessment. Significant *p* values (*p* < 0.05) are shown in bold.

### 3.3 Muscle MRI FF and Its Correlation With Clinical Functional Assessments

Among the 19 lower limb muscles studied, the gluteus maximus muscle had the highest FF values (32.3% [IQR 20.1%–48.9%]), followed by the adductor magnus muscle (22.4% [IQR 10.9%–49.0%]). Conversely, the adductor longus muscle showed the lowest FF values (2.5% [IQR 1.3%–7.5%]). For the leg muscles, the lateral head of gastrocnemius muscle (6.7% [IQR 3.6%–18.9%]) and the peroneal group muscles (6.1% [IQR 3.6%–14.7%]) exhibited the highest FF values. The tibialis posterior muscle showed the lowest FF values (2.1% [IQR 1.3%–3.6%]). Furthermore, the FF values for all muscles, except the tibialis posterior, were significantly higher in DMD patients compared to controls (*p* < 0.05).

**FIGURE 2:**
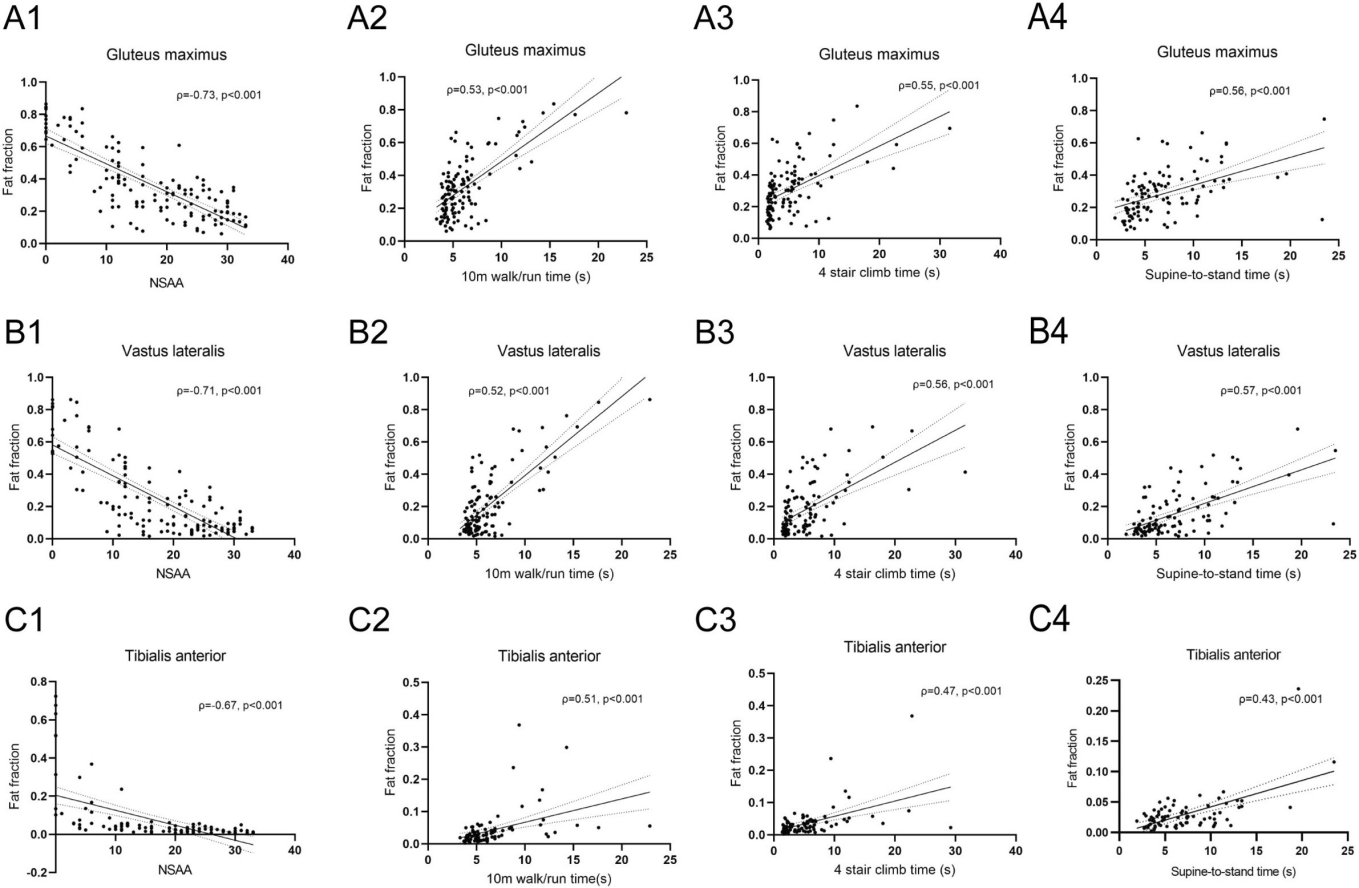
Relationship between muscle MRI FF and clinical functional assessments. (A1, B1 and C1) For NSAA, a pronounced negative correlation was observed with FF values of the gluteus maximus, vastus lateralis and tibialis anterior. (A2–4, B2–4 and C2–4) Timed function test durations exhibited a moderate positive correlation with FF values obtained from MR imaging. The regression lines were represented in black, and the 95% confidence intervals were delineated by dotted lines. FF, fat fraction; NSAA, North Star Ambulatory Assessment.

A significantly moderate to strong correlation was found between NSAA and FF of the 19 muscles (ρ = −0.32–−0.73, *p* < 0.001). For TFTs, most muscles showed a mild to moderate correlation (ρ = 0.27–0.60, *p* < 0.001), except the sartorius and gracilis muscles (Figure 2 and Table S5).

### 3.4 Spatial Distribution Pattern of Muscle Involvement

**FIGURE 3:**
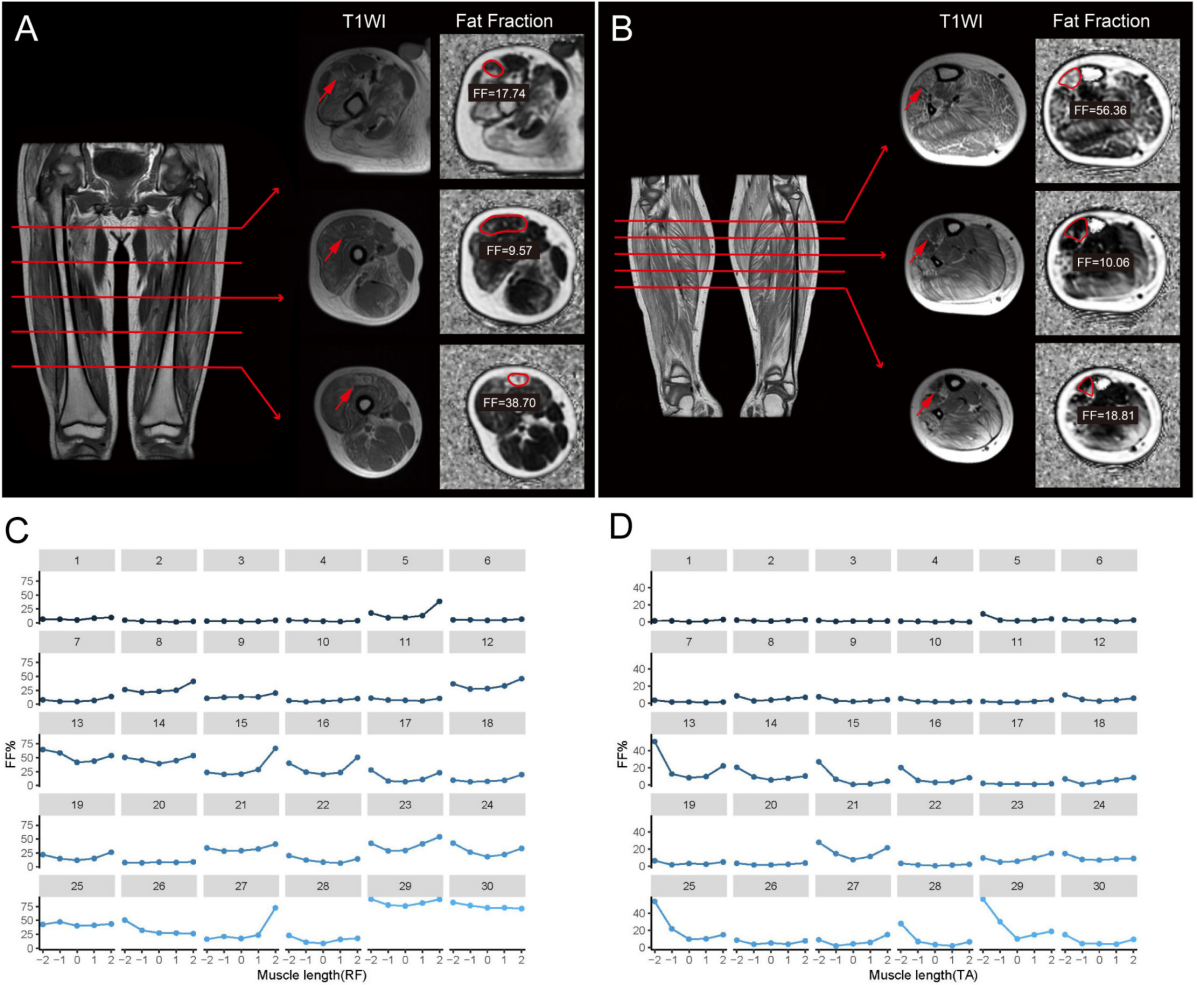
Fat distribution profiles along the full length of the muscles in DMD patients. For the (A) RF and (B) TA, three axial T1WI and IDEAL MRI slices were presented, elucidating the non‐uniform distribution of fat replacement along the proximodistal muscle axis of RF and TA. These two muscles were highlighted with manually drawn ROIs in IDEAL‐IQ and indicated with red arrows in T1WI. The coronal images provided an overview of the locations of the axial slices. (C,D) The diverse fat replacement patterns observed in RF and TA. The maxCSA was centred at ‘0’ on the *x*‐axis, with the proximal and mild‐proximal portions shown to the left (‘−2’, ‘−1’), and the distal and mild‐distal parts shown to the right (‘2’, ‘1’). Breakdown of the first 30 individual DMD patients was presented in each thumbnail plot. Filled circles represent FF values at different slices, connected by solid lines. A consistent muscle‐specific pattern of fat replacement was evident, observed in the majority. FF, fat fraction; maxCSA, maximum cross‐sectional area; RF, rectus femoris; ROI, region of interest; T1WI, T1‐weighted image; TA, tibialis anterior.

We observed significant differences in fatty infiltration across all eight muscles in the DMD group (*p* < 0.001) and five muscles in the control group when comparing the five MRI slices (Table S6). The inhomogeneous distribution of fatty infiltration across various spatial regions of each muscle was visualized (Figure 3 and Figure S3). Specific patterns became evident when comparing the proximal, middle and distal MRI slices of each muscle (Table S7). All muscles, except the tibialis posterior, exhibited higher FF values at the muscle ends compared to the muscle bellies. The rectus femoris and long head of biceps femoris muscles showed a typical U‐shaped FF distribution, with the highest value at the proximal and distal ends. The vastus lateralis, tibialis anterior and extensor digitorum longus muscles showed an UP‐type distribution, characterized by the highest FF values at only the proximal ends and the lowest at the centre regions. The peroneal group and soleus muscles showed a cubic function pattern, where FF values initially increased distally and then rose more proximally in later disease stages. In healthy controls, although the extensor digitorum longus and peroneal muscles showed inhomogeneous FF distribution, the differences were minimal (Tables S6 and S7 and Figure S4).

### 3.5 Temporal Distribution Pattern of Muscle Involvement

**FIGURE 4:**
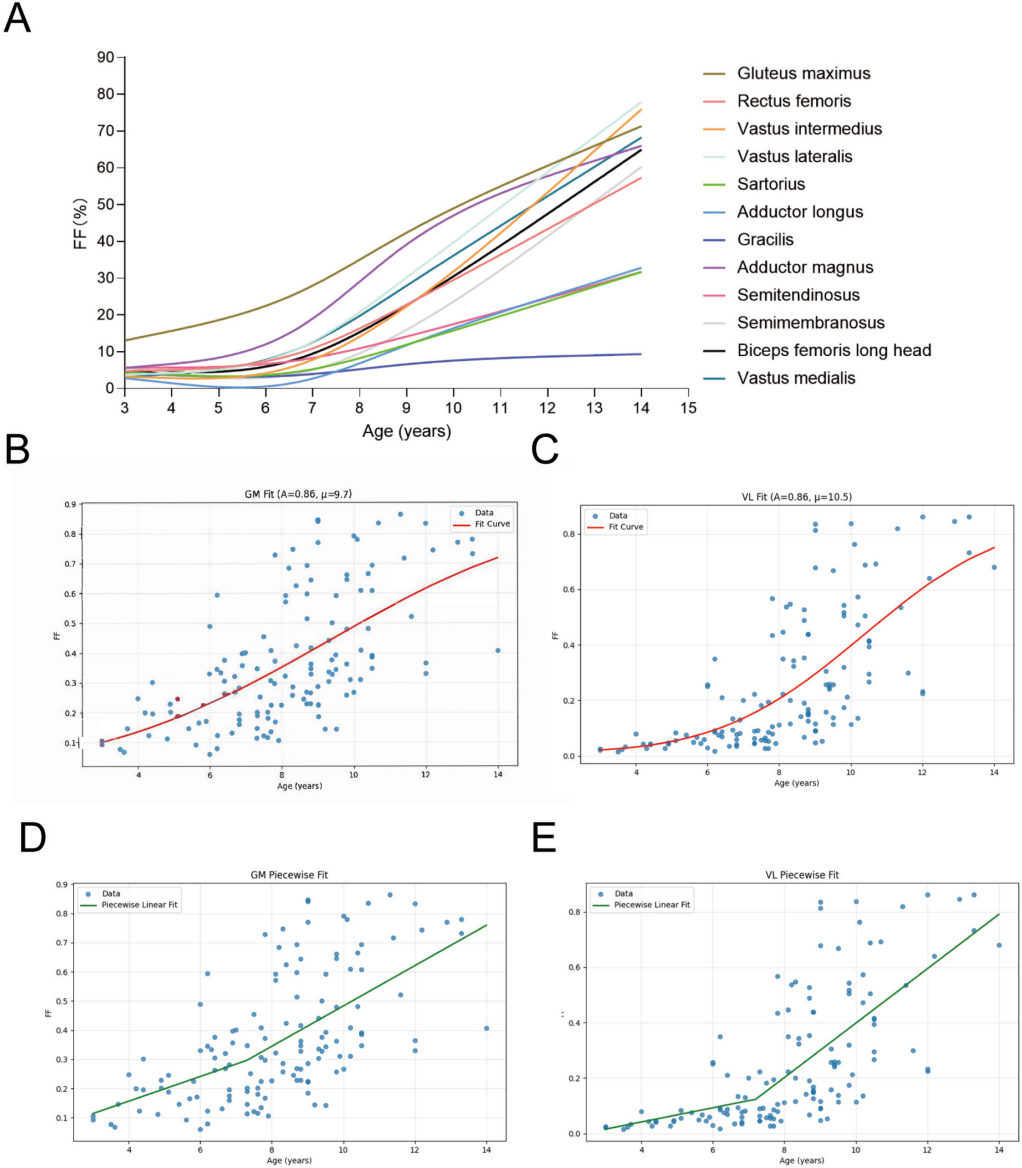
Population‐average modelling for MRI FF. (A) Progression of muscle fat replacement with increasing ages in DMD patients using three‐knot b‐splines reveals common inflection points and distinct progression patterns for thigh muscles. (B–E) Age‐associated changes in FF of the GM and VL muscles, with population‐level progression patterns estimated through NCDF model (B,C) and PL model (D–E). Filled circles denote FF values obtained from individual participants. Solid red and green lines show the NCDF model and PL model fit, respectively. FF, fat fraction; GM, gluteus maximus; NCDF, normal cumulative distribution function; PL, piecewise linear; VL, vastus lateralis.

We observed significant, inhomogeneous fatty infiltration that increased with age in each thigh and leg muscles (Figure 4A and Figure S5). The progression curves followed either a ‘slow‐fast’ or sigmoidal pattern, with an inflection point occurring around 7 years of age. Based on this observation and previous research [16, 18], we initially constructed a PL model (Equation 1) to calculate the average rate of fatty infiltration and the inflection point for each muscle. The average inflection point in DMD patients was 7.3 years (range, 5.2–7.6 years), with annual FF increases of 1.7% before and 6.7% after this point (Table 2). Figure 4D,E and Figure S5 show the distinct rates of annual fat accumulation in the gluteus maximus, vastus lateralis and tibialis anterior muscles.

**TABLE 2:** Population‐level PL model and NCDF model parameters for IDEAL‐IQ FF in thigh muscles of DMD and BMD group.

IDEAL‐IQ FF of muscles	PL model						NCDF model		
	DMD (*n* = 133)			BMD (*n* = 31)			DMD (*n* = 133)	BMD (*n* = 31)	*p*
	k_1_ (SD)	k_2_ (SD)	Age at x_0,_ y (SD)	k_1_ (SD)	k_2_ (SD)	Age at x_0,_ y (SD)	Age at 50% change in FF, μ, y (SD)	Age at 50% change in FF, μ, y (SD)	
Gluteus maximus	4.2 (2.3)	6.9 (1.2)	7.3 (2.1)	0.9 (0.9)	3.3 (0.6)	10.0 (2.3)	9.4 (1.8)	15.7 (0.9)	**0.003**
Vastus lateralis	2.6 (2.3)	9.8 (1.2)	7.2 (0.8)	0.2 (0.8)	2.7 (0.4)	8.9 (1.4)	10.5 (0.5)	16.6 (0.8)	**< 0.001**
Rectus femoris	1.7 (2.1)	7.0 (1.3)	7.3 (1.0)	0.2 (0.3)	1.0 (0.2)	10.2 (2.4)	11.8 (0.8)	22.1 (2.8)	**0.002**
Vastus intermedius	1.1 (2.2)	9.8 (1.1)	7.3 (0.6)	0.2 (1.0)	2.7 (0.4)	8.9 (1.7)	11.1 (0.4)	15.6 (0.5)	**< 0.001**
Vastus medialis	2.9 (2.0)	8.5 (1.1)	6.9 (0.9)	0.3 (0.7)	3.4 (0.4)	9.6 (1.3)	10.8 (0.7)	16.1 (0.9)	**< 0.001**
Sartorius	0.4 (1.4)	4.1 (0.9)	7.4 (1.0)	0.1 (0.3)	0.7 (0.2)	12.8 (3.9)	15.0 (1.0)	31.7 (4.5)	**0.002**
Gracilis	0.1 (1.0)	1.0 (0.4)	6.6 (2.2)	0.1 (0.2)	0.1 (0.1)	10.1 (2.3)	18.9 (4.1)	23.1 (3.9)	0.31
Adductor longus	0.3 (1.9)	4.7 (1.0)	7.0 (1.0)	0.1 (0.1)	0.1 (0.1)	9.5 (17.0)	13.7 (1.0)	35.4 (4.0)	**< 0.001**
Adductor magnus	4.3 (3.2)	8.1 (1.2)	6.6 (1.8)	0.4 (0.8)	3.9 (0.4)	9.1 (1.2)	9.0 (1.4)	16.6 (0.8)	**< 0.001**
Semitendinosus	0.8 (1.3)	3.7 (1.0)	7.6 (1.3)	0.2 (0.2)	1.7 (0.2)	12.5 (1.5)	15.0 (1.2)	15.0 (0.6)	0.06
Semimembranosus	0.2 (1.6)	8.4 (1.2)	7.6 (0.5)	0.4 (0.3)	4.8 (0.4)	13.4 (1.5)	12.1 (0.4)	17.6 (0.7)	**< 0.001**
Biceps femoris long head	1.5 (2.0)	8.3 (1.2)	7.4 (0.8)	0.2 (0.6)	4.2 (0.3)	10.0 (8.5)	11.7 (0.6)	16.1 (0.9)	**< 0.001**

*Note:* The parameters of PL model: k_1_ represents the slope of the first piece of the function; k_2_ is the slope of the second piece of the function; x_0_ represents the cut‐off value. The parameters of CDF model: μ represents the age of the greatest progression rate constant. The *p* values indicate the significance of difference in μ between BMD and DMD groups. Significant *p* values (*p* < 0.05) are shown in bold.

Abbreviations: NCDF, normal cumulative distribution function; PL, piecewise linear.

However, the PL model failed to capture the sigmoidal fatty infiltration patterns. Building on previous reports [15, 18], we constructed the NCDF model (Equation 2) to better characterize the sigmoidal pattern, positing that fatty deposit accumulation represents the temporal summation of the instantaneous fat accumulation rate. Figure 4B,C and Figure S5 present the population‐level NCDF modelling of the gluteus maximus, vastus lateralis and tibialis anterior muscles in DMD patients, where a progressive increase in μ values correlates with slower FF progression. The gracilis muscle had the highest μ (18.9 years) values among thigh muscles, indicating the slowest progression. The average μ for thigh muscles was 11.4 years (±2.8) (Table 2). These parameters align with findings from a previous longitudinal study [23].

**FIGURE 5:**
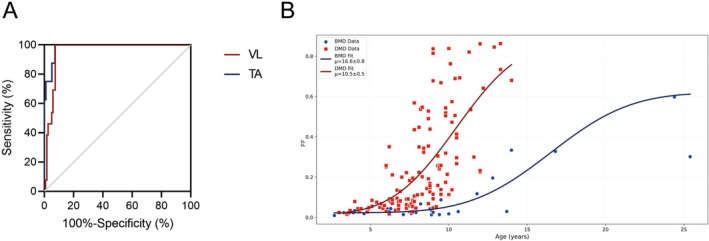
ROC curves for FF and population estimates for FF in DMD and BMD patients. (A) The ROC curve analysing FF and loss of ambulation for VL and TA showed an AUC of 0.956 and 0.983, respectively. Youden's index was 52.3% FF value for VL and 9.4% FF value for TA. (B) VL FF age trajectories for participants with DMD (red line, *n* = 133) and BMD (blue line, *n* = 31). The population estimated age at half‐maximum FF (μ) occurs 5.1 years earlier in VL in DMD compared to BMD. AUC, area under the curve; ROC, receiver operator characteristic.

We observed comparable performance among PL, NCDF1 and NCDF2 models with close AIC values in modelling disease progression through cross‐sectional FF data (Table S8). With 13 non‐ambulant patients in the DMD group, we constructed a receiver operating characteristic (ROC) curve and identified a 52.3% FF cut‐off in the vastus lateralis muscle for indicating loss of ambulation, achieving 92.5% sensitivity and 100% specificity (Figure 5A). In the BMD group, we applied both models and summarized the disease progression parameters in Table 2. The group averages showed slower progression in the BMD group across most muscles, with an average inflection age of 10.5 years and annual FF increases of 0.3% before the inflection point and 2.4% afterwards. In NCDF model, the average μ occurred 7.6 years later in BMD compared to DMD (Table 2). Figure 5B visualizes the representative FF trajectories for the vastus lateralis muscle, showing a 5.1‐year rightward shift (delayed μ values) in BMD trajectory relative to DMD.

## 4 Discussion

Our study confirmed the strong correlation of IDEAL‐IQ MRI FF measurements with histopathologic fatty infiltration and clinical functional assessments and integrated both temporal (age‐based modelling) and spatial (length‐specific) dimensions MRI data. This multimodal approach reinforces the utility of IDEAL‐IQ MRI in providing a comprehensive and robust evaluation of disease burden in DMD.

### 4.1 Muscle MRI Fat Fraction Acts as a Clinically Viable Alternative to Biopsy

Muscle MRI FF measurement yielded lower values than histopathology in muscles with mild fatty infiltration (< 20% FF) while exceeding biopsy values in severely affected muscles. This discrepancy is interesting and might indicate the methodological differences in fat quantification between MRI and histopathology. Specifically, MRI‐derived FF reflects the fraction of fat protons, whereas histopathological FF represents the volumetric proportion of fat within the tissue. This may lead to a pseudo‐overestimation of FF on MRI. De Wel et al. analysed FF in three different muscles (semimembranosus, vastus lateralis and rectus femoris) of limb girdle muscular dystrophy 12 patients and healthy controls using a 1.5‐T 6‐point Dixon (mDIXON Quant, Philips). They found a strong correlation between MRI and biopsy FF, with MRI FF values generally being higher. Notably, the correlation was not significant in control subjects or in the rectus femoris muscles of patients, partly due to the relatively lower FF in these muscles. In our data, the MRI FF in healthy controls ranged from 0.1% to 21.3%. These results suggest that MRI‐derived FF values may be more variable and less reliable in muscles with minimal fatty infiltration.

Although IDEAL‐IQ MRI‐derived FF correlates well with biopsy FF (ρ = 0.98), such findings should be interpreted with caution. A previous study showed that FF values using the Philips platform were slightly higher than with GE systems [24]. Although direct comparisons between various platforms were not performed in our study, the relatively lower FF values obtained using IDEAL‐IQ in the previous study [24], coupled with the generally higher FF on MRI compared to histology, may offset each other and result in a closer alignment with histological quantification.

Fatty infiltration of muscles is increasingly used for monitoring disease progression and evaluating treatment response in DMD [9, 25]. Although muscle biopsy remains the gold standard for quantifying fatty infiltration [17], its invasiveness and limited sampling restrict representation of disease heterogeneity [26]. Previous studies have established the accuracy and reproducibility of FF determination on MRI using lipid emulsions [27, 28]. Smith et al. evaluated FF using a 2‐point Dixon at 1.5 T and 3 T on eight muscle samples from pigs and rabbits. They found no significant difference between histological and imaging results [29]. In addition, emerging studies have validated Dixon‐based MRI FF against histology across neuromuscular disorders [12, 17, 29, 30, 31, 32], consistently demonstrating strong correlations despite systematic overestimation by MRI. This discrepancy may be due to technical factors (MRI device/field strength variations) or biological differences (disease cohorts or inclusion of intramyocytic lipids in MRI) [12, 24]. Our results and findings in the previous studies [12, 17, 27, 28, 29, 30, 31, 32] together indicate that quantitative MRI and FF measurements can act as an alternative to muscle biopsy for quantifying the fatty infiltration.

### 4.2 Fatty Infiltration Patterns and Muscle‐Specific FF Correlations With Clinical Function

Consistent with previous DMD studies [33], we observed the highest FF in the gluteus maximus and adductor magnus muscles, followed by the quadriceps and biceps femoris muscles. Although most existing literature focuses on pelvic and thigh muscles, our findings highlight notable involvement of lower leg muscles, particularly the gastrocnemius and peroneal groups, which were more severely affected than the sartorius, gracilis and adductor longus muscles of the thigh [34]. A recent study revealed no significant difference in MRI T2 values between the deltoid, biceps brachii and triceps brachii muscles and the soleus, medial gastrocnemius and peroneal muscles across DMD age groups [35], demonstrating concurrent fatty infiltration in both arm and leg muscles. Although data on arm muscles were not available, making a direct comparison impossible, these findings may suggest that at certain stages of the disease, previously preserved thigh muscles, weight‐bearing leg muscles and functionally active arm muscles could undergo concurrent fatty infiltration. Critically, we found lateral‐posterior‐predominant leg fatty infiltration, which is consistent with the previous findings in BMD and *DMD* female carriers [36, 37].

Clinical assessments such as the 6‐min walk test (6MWT), TFTs, motor function measure and NSAA scales are validated for evaluating motor impairment in muscular dystrophies [38]. Barnard et al. conducted longitudinal analysis with FF determined by ^1^H MRS and observed a linear decline in function measures as vastus lateralis FF increased [23]. Despite differences in MRI acquisition and analysis method, we found that FF in most muscles showed moderate to strong correlations with functional assessments, consistent with the findings in both a previous small‐cohort thigh muscle study and a large‐cohort upper extremity study [9, 35]. However, we did not observe a significant correlation between FF and TFTs in the gracilis muscle, likely due in part to its relatively lower FF values. Interestingly, other muscles with similarly slight fatty infiltration—such as the adductor longus (median FF 2.5%) and tibialis anterior (median FF 3.0%)—still demonstrated moderate correlations with both NSAA scores and TFTs. These findings suggest that certain muscles may serve as more sensitive indicators of disease progression than others.

### 4.3 Non‐Uniform Longitudinal Fat Distribution Reflects Anatomical/Mechanical Determinants and Disease‐Stage Heterogeneity

We observed non‐uniform fatty infiltration along the length of specific muscles, which was most pronounced in the intermediate and late stages of the disease. In our study, the tibialis anterior and extensor digitorum longus muscles exhibited the highest FF values at the proximal ends, consistent with previous findings [39]. Hooijmans et al. using quantitative 3‐point Dixon MRI and five non‐consecutive slices over a length of up to 13 cm revealed higher fat fractions in both the distal and proximal segments of seven muscles in patients with DMD. They also found that a 15‐mm shift in measurement location could result in a difference in mean FF ranging from 1% to 12% [11]. The authors suggested that the myotendinous junctions of these muscles experience the highest mechanical strain. Furthermore, dystrophin is particularly concentrated near these end regions, rendering dystrophin‐deficient muscles more susceptible to injury.

Although mechanical disruption is a contributing factor, our study found that certain muscles had significantly higher FF at the insertion or origin, whereas others displayed such variability among participants that no consistent pattern emerged. Muscle anatomy, including shape and biarticular/monoarticular architecture, affects the distribution of mechanical stress [40, 41]. We found that biarticular muscles such as the rectus femoris and biceps femoris muscles demonstrated greater variability among patients. Additionally, heterogeneity in genetic expression profiles, embryological origin, metabolism, cell population and even non‐contractile tissue may contribute to differences in the severity and pattern of fat replacement [42]. A study suggested that muscles with longer fibres and larger physiological cross‐sectional areas are preferentially affected in DMD [41]. Unlike a previous study [12], we did not observe a downward parabola pattern (highest FF at the centre), potentially due to specific disease characteristics. Further research is warranted to explore other aetiologies underlying diverse fat replacement patterns and to ascertain potential correlations with serum creatine kinase levels, abnormal postures (like foot drop) and Achilles tendon contracture.

### 4.4 IDEAL‐IQ FF Modelling Reveals MRI Biomarkers of Disease Trajectories

We used cross‐sectional IDEAL‐IQ data of lower limb muscles and age to apply PL and NCDF models, providing a detailed quantification of disease progression in DMD. In the study by Rooney et al. [15], the parameters A and C were fixed among various muscles (the NCDF model), assuming that the maximum and initial values of each muscle were the same. We posit that different muscles may have varying maximum values due to factors such as mechanical stress and muscle anatomy, as discussed in the spatial distribution section. Therefore, in our study, rather than using fixed parameters, we determined A either by parameter estimation or based on the population distribution of FF values. Despite variability in muscle involvement and progression at the individual level, as well as differing methods of parameter calculation [15], our study showed that the NCDF model provided good fits to the cross‐sectional MRI data at the population level. The average μ and σ values were highly consistent with those reported in longitudinal studies.

Using the PL model, we observed a clear point of slope change at approximately 7 years in patients with DMD, consistent with previous findings in functional domains, including NSAA and 6MWT results, as well as muscle ultrasound measures [43, 44]. Although the PL model is simpler, it demonstrates comparable performance to the NCDF model. Similar to a previous study [15], both the PL and NCDF models revealed a faster initial increase and slower midterm progression in the gluteus maximus compared to the vastus lateralis muscle, suggesting that the gluteus maximus muscle experiences primary injury and fat replacement early (< 7 years), whereas the vastus lateralis muscle progresses later (> 7 years).

Naarding et al. [18] observed a sigmoidal progression of FF in the VL muscle. Their longitudinal MRI scans revealed that a 10% increase in FF was associated with a 4.11‐fold increase in the instantaneous risk of LoA in DMD patients. Our data further identify 52.3% VL FF as a highly sensitive and specific threshold for indicating LoA. Collectively, these findings suggest that VL FF may serve as a robust quantitative biomarker in DMD. The extended age inflection points in the PL model (10.5 years in BMD vs. 7.3 years in DMD) and the delayed μ emergence in NCDF model (occurring 7.6 years later in BMD than DMD) collectively demonstrate distinct progression patterns between DMD and BMD.

## 5 Limitations

The present study has several limitations. Our analysis identified heterogeneous fatty infiltration patterns, yet clinical correlations were assessed at single cross‐sectional levels. This sampling approach risks bias in whole‐muscle fat estimation, potentially explaining differential correlations between individual muscles and clinical indicators. Moreover, unlike the comprehensive 3D modelling approach adopted in a previous study [12] that captured continuous longitudinal distribution patterns through whole‐muscle analysis, our reliance on five discontinuous slices may have obscured certain spatial configurations.

Second, although group‐level differences in fat fraction progression between DMD and BMD were identified, the cross‐sectional study design precludes characterization of individual longitudinal variation. The relatively young age of our cohort and the limited number of LoA events may compromise the vastus lateralis cut‐off accuracy for indicating LoA. Additionally, lower FF levels in leg muscles and lack of advanced‐stage data precluded adequate fitting of NCDF and PL models for most leg muscles. Nevertheless, these data provide progression metrics across multiple thigh muscles. Finally, this study did not include direct comparisons between IDEAL‐IQ and other Dixon‐based techniques, which limits our ability to determine whether observed differences with other studies arise from technique‐specific factors versus sampling effects. This implies that caution should be exercised when interpreting these results as indicative of IDEAL‐IQ's superior performance relative to other sequences.

In conclusion, our study demonstrates a significant relationship between FF on IDEAL‐IQ MRI and histopathologic muscle fatty infiltration, as well as ambulatory clinical function in DMD. These findings support the potential utility of IDEAL‐IQ MRI FF as non‐invasive imaging biomarkers for evaluating disease progression in DMD. Furthermore, the distinct inhomogeneous distribution pattern of fatty infiltration observed in the lower limb muscles underscores the importance of reproducible limb positioning in future longitudinal studies to minimize the impact of spatial heterogeneity in muscle damage. The construction of two disease progression models illustrates the temporal patterns of population‐level FF changes across the dystrophinopathy spectrum, including both DMD and BMD patients.

## Funding

This work was supported by the National High‐Level Hospital Clinical Research Funding (High‐Quality Clinical Research Project of Peking University First Hospital; grant number 2023HQ10) and the National Natural Science Foundation of China (grant number 82201553) and Peking University Clinical Scientist Training Program (BMU2025PYJH039).

## Ethics Statement

The study was approved by the Ethics Committee of Peking University First Hospital in accordance with the Declaration of Helsinki and its later amendments. Written informed consent was obtained from all adult patients or parents/guardians of minors. The authors of this manuscript certify that they comply with the ethical guidelines for authorship and publishing in the *Journal of Cachexia, Sarcopenia and Muscle*.

## Conflicts of Interest

The authors declare no conflicts of interest.

## Supporting information


**Table S1:** Slice selections for measuring specific skeletal muscles.
**Table S2:** Comparison of muscle MRI FF and histopathological fatty infiltration percentage.
**Table S3:** Reproducibility of MRI measurement in three observers.
**Table S4:** Consistency of MRI measurement between three observers.
**Table S5:** Correlation between fat fraction of MRI and ambulatory function.
**Table S6:** Differences in FF values of thigh and calf muscles along the length axis.
**Table S7:** Analysis of inhomogeneity of fat distribution over the length of thigh muscles.
**Table S8:** The comparison between PL model and NCDF model in DMD and BMD group.
**Figure S1:** Overview of participant enrolment and study design.
**Figure S2:** Four selected slices for ROI placement of different muscle compartments.
**Figure S3:** Fat distribution profiles along the length of muscles in DMD patients.
**Figure S4:** Profiles of fat distribution along the length of controls.
**Figure S5:** Population‐average modelling for MRI FF for leg muscles.
