# Supplementary material for: Quantitative MRI Findings and Their Relationship to Muscle Histopathology and Ambulatory Clinical Function in Duchenne Muscular Dystrophy
Source: J Cachexia Sarcopenia Muscle. 2026 Jan 25;17(1):e70205. doi: 10.1002/jcsm.70205 (PMC12833498; doi:10.1002/jcsm.70205)

**Supplementary materials**

**Supplementary Tables**

**Table S1** Slice selections for measuring specific skeletal muscles

| **Slice No.** | **Position** | **Muscles** |
| --- | --- | --- |
| 1 | Greater trochanter | Gluteus maximus |
| 2 | Proximal 1/3 of the thigh | Rectus femoris, Vastus lateralis, Vastus intermedius, Sartorius, Gracilis, Adductor magnus, Adductor longus, Semitendinosus |
| 3 | Distal 1/3 of the thigh | Vastus medialis, Semimembranosus, Biceps femoris long head |
| 4 | Maximum cross section of the calf | Tibialis anterior, Extensor digitorum longus, Peroneal group, Tibialis posterior, Soleus, Medial head of gastrocnemius, Lateral head of gastrocnemius |

**Table S2** Comparison of muscle MRI FF and histopathological fatty infiltration percentage

| **Patient No.** | **FF (%) on MRI** | **Histopathological fatty infiltration (%)** | **Difference** |
| --- | --- | --- | --- |
| 1 | 1.46 | 2.07 | -0.61 |
| 2 | 2.25 | 6.64 | -4.39 |
| 3 | 3.56 | 4.09 | -0.53 |
| 4 | 4.02 | 7.26 | -3.24 |
| 5 | 15.87 | 13.34 | 2.53 |
| 6 | 17.38 | 22.90 | -5.52 |
| 7 | 22.23 | 19.03 | 3.20 |
| 8 | 34.32 | 30.44 | 3.88 |
| 9 | 51.76 | 56.38 | -4.62 |
| 10 | 78.83 | 71.12 | 7.71 |

The median muscle MRI FF and histopathological fatty infiltration percentage was 16.63% (3.23%–38.68%) and 16.19% (6.00%–36.93%), respectively. Compared with biopsy, the mean paired difference with IDEAL-IQ MRI was -0.16 ± 4.39. FF, fat fraction; MRI, magnetic resonance imaging.

**Table S3** Reproducibility of MRI measurement in three observers

| **Groups** | **ICC** | **95% CI** | ***P* value** |
| --- | --- | --- | --- |
| **A_1_-A_2_** | 0.996 | 0.989–0.999 | 0.216 |
| **B_1_-B_2_** | 0.993 | 0.981–0.997 | 0.321 |
| **C_1_-C_2_** | 0.994 | 0.983–0.998 | 0.241 |

A, B, and C represent the three observers. The numbers (1, and 2) at the subscript represent the order of assessments. ICC, intraclass correlation coefficient.

**Table S4** Consistency of MRI measurement between three observers

| **Groups** | **ICC** | **95% CI** | ***P* value** |
| --- | --- | --- | --- |
| **A_1_-B_1_** | 0.991 | 0.976–0.997 | 0.812 |
| **A_1_-C_1_** | 0.995 | 0.986–0.998 | 0.950 |
| **B_1_-C_1_** | 0.991 | 0.977–0.997 | 0.899 |

**Table S5** Correlation between fat fraction of MRI and ambulatory function

|  | **NSAA** | | **10m walk/run** | | **4 stair climb** | | **Supine-to-stand** | |
| --- | --- | --- | --- | --- | --- | --- | --- | --- |
|  | **ρ** | ***P* value** | **ρ** | ***P* value** | **ρ** | ***P* value** | **ρ** | ***P* value** |
| **Pelvic girdle and Thigh** | n = 133 | | n = 120 | | n = 114 | | n = 103 | |
| **Gluteus maximus** | -0.73 | **<0.001** | 0.53 | **<0.001** | 0.55 | **<0.001** | 0.56 | **<0.001** |
| **Rectus femoris** | -0.69 | **<0.001** | 0.48 | **<0.001** | 0.51 | **<0.001** | 0.50 | **<0.001** |
| **Vastus intermedius** | -0.71 | **<0.001** | 0.49 | **<0.001** | 0.54 | **<0.001** | 0.56 | **<0.001** |
| **Vastus lateralis** | -0.72 | **<0.001** | 0.52 | **<0.001** | 0.56 | **<0.001** | 0.57 | **<0.001** |
| **Vastus medialis** | -0.68 | **<0.001** | 0.49 | **<0.001** | 0.55 | **<0.001** | 0.60 | **<0.001** |
| **Sartorius** | -0.45 | **<0.001** | 0.19 | **0.04** | 0.16 | 0.09 | 0.27 | **0.006** |
| **Gracilis** | -0.32 | **<0.001** | 0.08 | 0.41 | -0.003 | 0.97 | 0.17 | 0.09 |
| **Adductor magnus** | -0.69 | **<0.001** | 0.47 | **<0.001** | 0.54 | **<0.001** | 0.55 | **<0.001** |
| **Adductor longus** | -0.55 | **<0.001** | 0.31 | **<0.001** | 0.32 | **<0.001** | 0.37 | **<0.001** |
| **Semitendinosus** | -0.53 | **<0.001** | 0.28 | **0.002** | 0.29 | **0.002** | 0.28 | **0.005** |
| **Semimembranosus** | -0.56 | **<0.001** | 0.32 | **<0.001** | 0.34 | **<0.001** | 0.38 | **<0.001** |
| **Biceps femoris long head** | -0.59 | **<0.001** | 0.37 | **<0.001** | 0.41 | **<0.001** | 0.44 | **<0.001** |
| **Leg** | n = 105 | | n = 96 | | n = 90 | | n = 79 | |
| **Tibialis anterior** | -0.68 | **<0.001** | 0.54 | **<0.001** | 0.47 | **<0.001** | 0.43 | **<0.001** |
| **Extensor digitorum longus** | -0.62 | **<0.001** | 0.51 | **<0.001** | 0.35 | **<0.001** | 0.31 | **0.006** |
| **Peroneal group** | -0.65 | **<0.001** | 0.56 | **<0.001** | 0.46 | **<0.001** | 0.47 | **<0.001** |
| **Tibialis posterior** | -0.56 | **<0.001** | 0.45 | **<0.001** | 0.39 | **<0.001** | 0.41 | **<0.001** |
| **Soleus** | -0.67 | **<0.001** | 0.52 | **<0.001** | 0.41 | **<0.001** | 0.33 | **0.003** |
| **Medial head of gastrocnemius** | -0.65 | **<0.001** | 0.51 | **<0.001** | 0.45 | **<0.001** | 0.42 | **<0.001** |
| **Lateral head of gastrocnemius** | -0.61 | **<0.001** | 0.47 | **<0.001** | 0.38 | **<0.001** | 0.34 | **0.002** |

Overview of Spearman correlation coefficients (ρ) for MRI fat fraction of the respective muscles and the ambulatory clinical function measures in DMD patients. Significant *P* values (*P* < 0.05) are marked in bold. NSAA: North Star Ambulatory Assessment; MRI, magnetic resonance imaging.

**Table** **S6** Differences in FF values of thigh and calf muscles along the length axis

|  | **DMDs** | | | | | | **Controls** | | | | | |
| --- | --- | --- | --- | --- | --- | --- | --- | --- | --- | --- | --- | --- |
|  | **-2** | **-1** | **0** | **1** | **2** | ***P* value** | **-2** | **-1** | **0** | **1** | **2** | ***P* value** |
| **Rectus femoris** | 29.00±22.90 | 22.72±20.86 | 20.19±18.69 | 22.73±19.60 | 31.89±22.46 | **<0.001** | 1.43±0.78 | 1.22±0.75 | 1.21±1.13 | 1.71±1.72 | 1.37±1.28 | **0.012** |
| **Vastus lateralis** | 41.45±26.08 | 33.44±25.70 | 27.40±21.62 | 24.98±19.40 | 32.65±21.56 | **<0.001** | 1.67±0.77 | 1.52±0.59 | 1.90±0.90 | 1.94±1.31 | 2.34±1.70 | **0.007** |
| **Biceps femoris long head** | 26.84±19.84 | 23.47±20.32 | 22.23±21.35 | 23.68±21.40 | 25.20±20.83 | **<0.001** | 2.75±1.95 | 2.69±1.56 | 2.49±1.70 | 2.53±1.65 | 2.69±1.73 | 0.458 |
| **Tibialis anterior** | 13.93±15.30 | 5.29±6.55 | 3.45±2.74 | 4.26±3.68 | 7.32±5.99 | **<0.001** | 2.00±1.02 | 1.28±0.72 | 1.44±0.85 | 1.36±0.98 | 1.58±1.46 | **0.002** |
| **Extensor digitorum longus** | 11.29±11.81 | 6.19±7.44 | 5.25±6.91 | 6.42±8.10 | 8.21±9.00 | **<0.001** | 2.25±0.99 | 1.78±1.10 | 1.81±1.07 | 1.75±0.91 | 1.49±0.75 | **0.006** |
| **Peroneal group** | 15.42±17.46 | 11.00±11.26 | 13.01±12.59 | 19.26±16.31 | 20.48±17.03 | **<0.001** | 2.63±1.49 | 2.52±1.93 | 3.19±2.57 | 2.59±1.64 | 2.07±1.27 | **0.002** |
| **Tibialis posterior** | 3.10±2.35 | 2.06±1.58 | 2.76±2.51 | 2.78±1.94 | 3.47±2.51 | **<0.001** | 1.48±0.77 | 1.37±0.62 | 1.66±0.72 | 1.53±0.64 | 1.37±0.77 | 0.198 |
| **Soleus** | 8.53±8.34 | 9.16±9.26 | 10.17±9.34 | 11.86±10.71 | 15.07±13.36 | **<0.001** | 2.37±0.73 | 2.33±0.71 | 2.52±0.84 | 2.49±0.80 | 2.35±0.86 | 0.105 |

Friedman test was used to compare the ordinal muscles. For all muscles of patients, the differences were significantly superior at *P* < 0.001, which mean that the measured fat fractions were not homogeneously distributed along the length of all the individual muscles in DMD. FF, fat fraction; DMD, Duchenne muscular dystrophy.

**Table S7** Analysis of inhomogeneity of fat distribution over the length of thigh muscles

|  | **DMDs** | | | | | | | **Controls** | | | | | | |
| --- | --- | --- | --- | --- | --- | --- | --- | --- | --- | --- | --- | --- | --- | --- |
|  | **FF_-2_-FF_0_** | ***P* value** | **FF_2_-FF_0_** | ***P* value** | **FF_-2_-FF_2_** | ***P* value** | **Pattern** | **FF_-2_-FF_0_** | ***P* value** | **FF_2_-FF_0_** | ***P* value** | **FF_-2_-FF_2_** | ***P* value** | **Pattern** |
| **Rectus femoris** | 8.81±8.30 | **<0.001** | 11.70±10.90 | **<0.001** | -2.89±11.46 | 0.087 | U | 0.23±1.30 | **0.030** | 0.16±0.58 | 0.21 | 0.06±1.43 | 0.100 | undefined |
| **Vastus lateralis** | 14.05±9.46 | **<0.001** | 5.25±8.77 | **0.001** | 8.80±11.80 | **0.001** | UP | -0.23±0.81 | 0.135 | 0.45±0.96 | **0.041** | -0.68±1.50 | **0.008** | undefined |
| **Biceps femoris long head** | 4.61±6.18 | **<0.001** | 2.97±7.28 | **0.002** | 1.64±7.81 | 0.444 | U | 0.26±0.68 | **0.043** | 0.20±0.79 | 0.286 | 0.06±0.98 | 0.349 | undefined |
| **Tibialis anterior** | 10.48±13.20 | **<0.001** | 3.87±3.78 | **<0.001** | 6.61±11.38 | **0.002** | UP | 0.56±0.86 | **0.001** | 0.14±1.04 | 0.726 | 0.42±1.26 | **0.040** | undefined |
| **Extensor digitorum longus** | 6.04±8.63 | **<0.001** | 2.96±4.29 | **<0.001** | 3.09±8.84 | **0.008** | UP | 0.44±0.88 | **0.009** | -0.32±0.94 | 0.162 | 0.76±0.95 | **0.001** | FP |
| **Peroneal group** | 2.41±12.43 | 0.750 | 7.48±10.19 | **<0.001** | -5.06±9.50 | **0.001** | FD | -0.54±1.45 | 0.112 | -1.10±1.62 | **<0.001** | 0.56±0.83 | **0.006** | FP |
| **Tibialis posterior** | 0.34±2.11 | 0.292 | 0.71±1.27 | **0.002** | -0.37±2.01 | 0.053 | undefined | -0.18±0.90 | 0.389 | -0.29±0.65 | **0.031** | 0.10±0.82 | 0.679 | undefined |
| **Soleus** | -1.63±2.72 | **<0.001** | 4.91±6.56 | **<0.001** | -6.54±7.52 | **<0.001** | FD | -0.16±0.45 | 0.074 | -0.18±0.55 | 0.088 | 0.02±0.60 | 0.426 | undefined |

Wilcoxon signed rank test was used to compare the differences of proximal versus middle, distal versus middle, and proximal versus distal FF values. We described a rough pattern for proximo-distal fat replacement in each muscle that was followed by majority of patients and controls: an upward facing parabola (“U”), an upward facing parabola with proximal prominent (“UP”), a cubic function with proximal or distal prominent (“FP” or “FD”) and an “undefined” pattern (with little coherence among subjects). *P* <0.05 was considered significant. FF, fat fraction; DMD, Duchenne muscular dystrophy.

Table S8 The comparison between PL model and NCDF model in DMD and BMD group

| **Muscles** | **DMD** | | | | **BMD** | | | |
| --- | --- | --- | --- | --- | --- | --- | --- | --- |
|  | **AIC_NCDF1** | **AIC_NCDF2** | ***P* value** | **AIC_PL** | **AIC_NCDF1** | **AIC_NCDF2** | ***P* value** | **AIC_PL** |
| **Gluteus maximus** | -467.34 | -470.77 | 0.753 | -466.9 | -160.36 | 148.18 | **<0.001** | **-158.4*** |
| **Vastus lateralis** | -464.42 | -468.38 | 0.984 | -465.6 | -170.38 | 162.59 | **0.003** | -168.4 |
| **Rectus femoris** | -463.47 | -467.08 | 0.822 | -464.3 | -218.22 | 217.61 | **0.100** | -216.2 |
| **Vastus intermedius** | -476.08 | -480.08 | 0.997 | -476.5 | -175.58 | 155.06 | **<0.001** | **-173.6*** |
| **Vastus medialis** | -493.0 | -498.82 | 0.920 | -496.2 | -188.25 | 162.58 | **<0.001** | **-186.3*** |
| **Sartorius** | -562.13 | -562.13 | 0.337 | -561.8 | -231.04 | 218.11 | **<0.001** | -221.7 |
| **Gracilis** | -745.64 | -746.33 | 0.191 | -743.6 | -223.74 | 213.59 | **<0.001** | **-251.0*** |
| **Adductor longus** | -512.85 | -512.83 | 0.134 | -511.9 | -273.14 | 267.52 | **<0.001** | -271.1 |
| **Adductor magnus** | -431.45 | -433.25 | 0.333 | -432.2 | -163.52 | 157.16 | **0.006** | -161.5 |
| **Semitendinosus** | -550.70 | -553.62 | 0.583 | -550.8 | -231.04 | 218.11 | **<0.001** | **-229.0*** |
| **Semimembranosus** | -497.03 | -498.83 | 0.333 | -497.0 | -196.85 | -189.2 | **0.003** | -194.9 |
| **Biceps femoris long head** | -469.05 | -472.05 | 0.608 | -468.9 | -184.79 | 162.58 | **<0.001** | **-182.8*** |

NCDF1 represents a four-parameter model, in which A, μ, σ, and C are all parameters that are optimized by the model. NCDF2 represents a two-parameter model that retains only μ and σ as model parameters, with A derived from the maximum value in the current muscle data and c set to 0.01. A lower AIC value indicates a superior model. NCDF1 and NCDF2 are nested models, and the likelihood ratio test is used for comparison, with *P* < 0.05 indicating a statistically significant difference. NCDF2 and the PL model are two independent models that are directly compared using ΔAIC, calculated as ΔAIC = AIC(CDF1 model)–AIC(PL model). A positive value indicates that the PL model is superior, while a negative value suggests that the NCDF1 model is more favorable. Asterisks (*) denote |ΔAIC| > 10, indicating a significant difference.

**Supplementary Figures**

**Figure S1** Overview of participant enrollment and study design

**
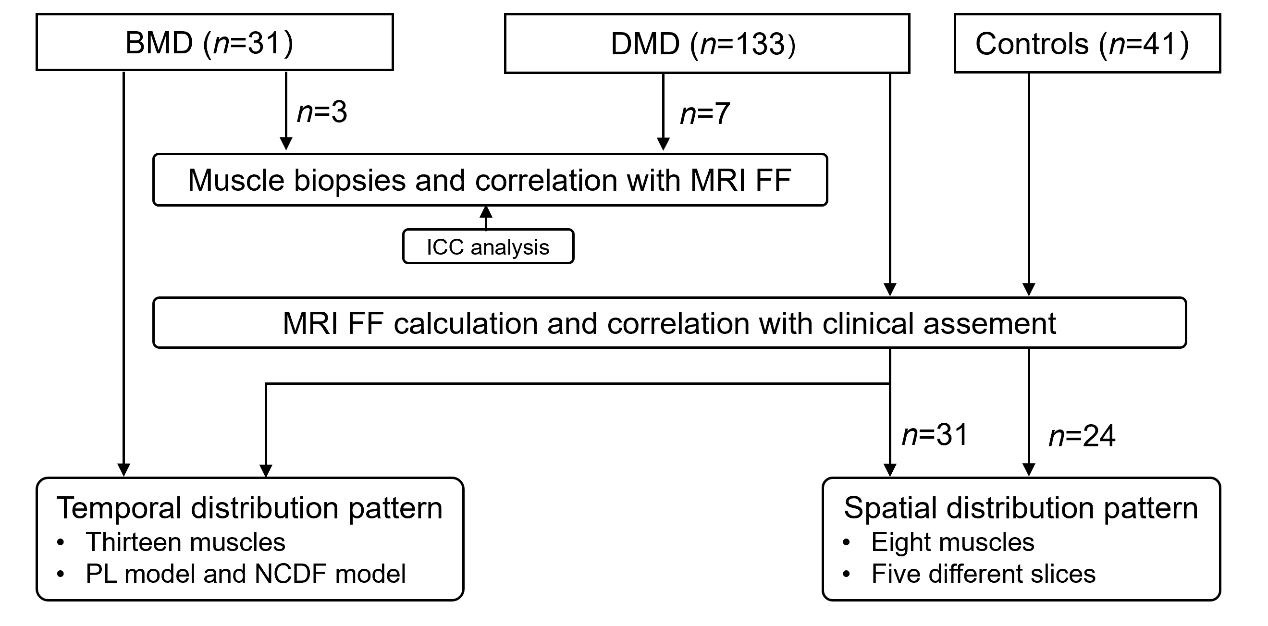
**

FF, fat fraction. DMD, Duchenne muscular dystrophy. BMD, Becker muscular dystrophy. ICC, intraclass correlation coefficient. PL, piecewise linear. NCDF, piecewise linear.

**Figure S2** Four selected slices for ROI placement of different muscle compartments


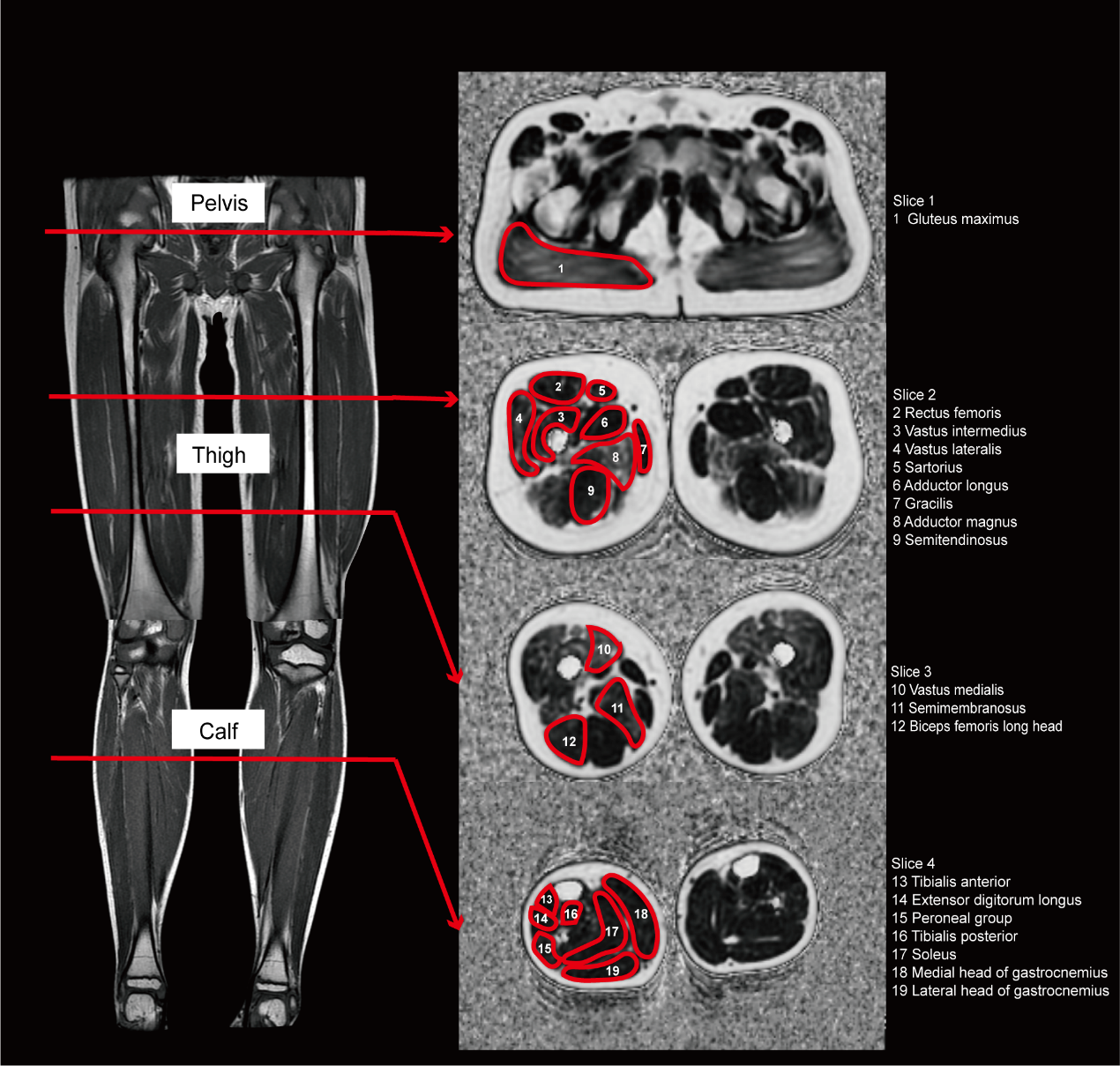


ROI, region of interest.


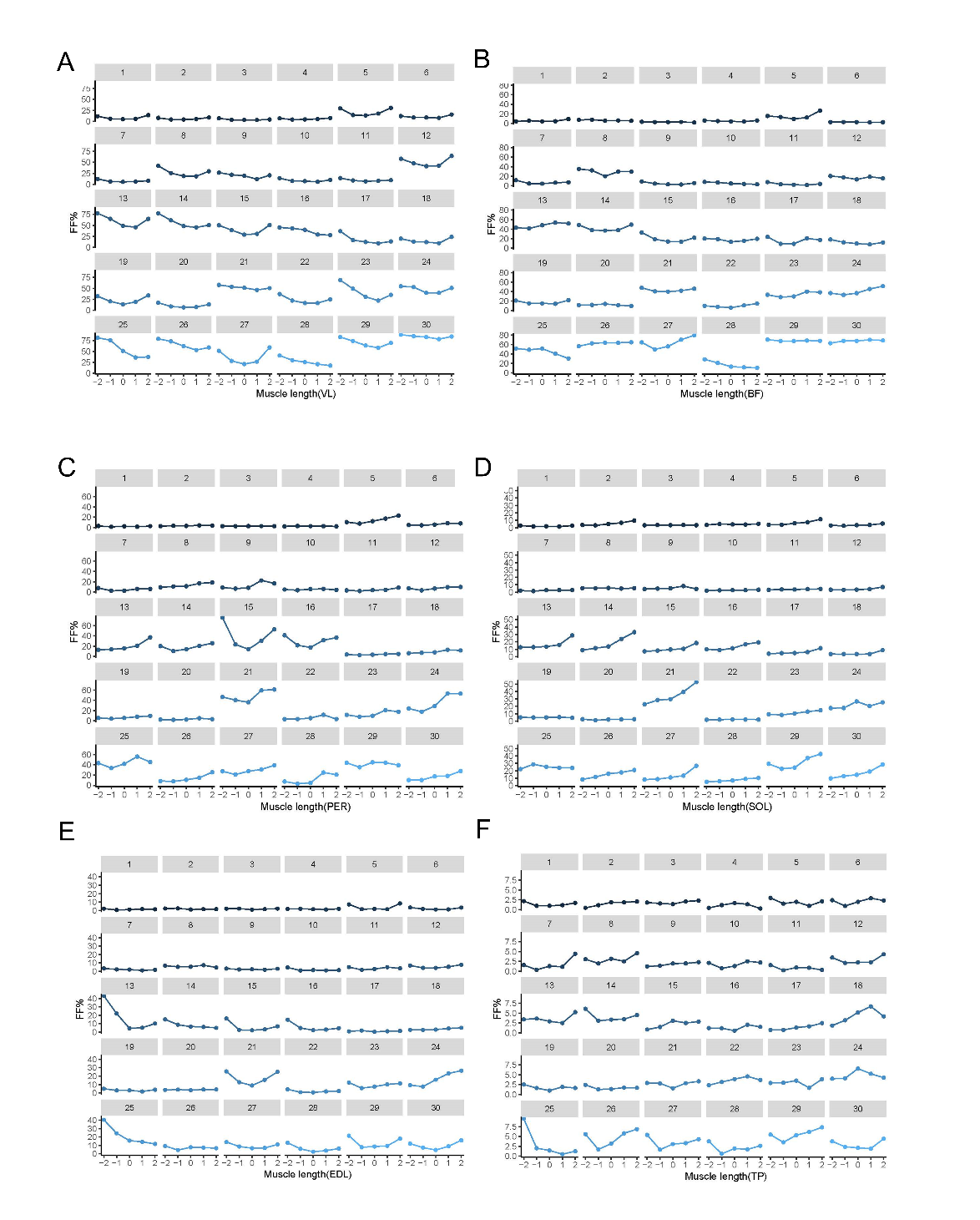
**Figure S3** Fat distribution profiles along the length of muscles in DMD patients

RF, rectus femoris; BF, biceps femoris long head; TP, tibialis posterior; EDL, extensor digitorum longus; DMD, Duchenne muscular dystrophy.

**Figure S4** Profiles of fat distribution along the length of controls


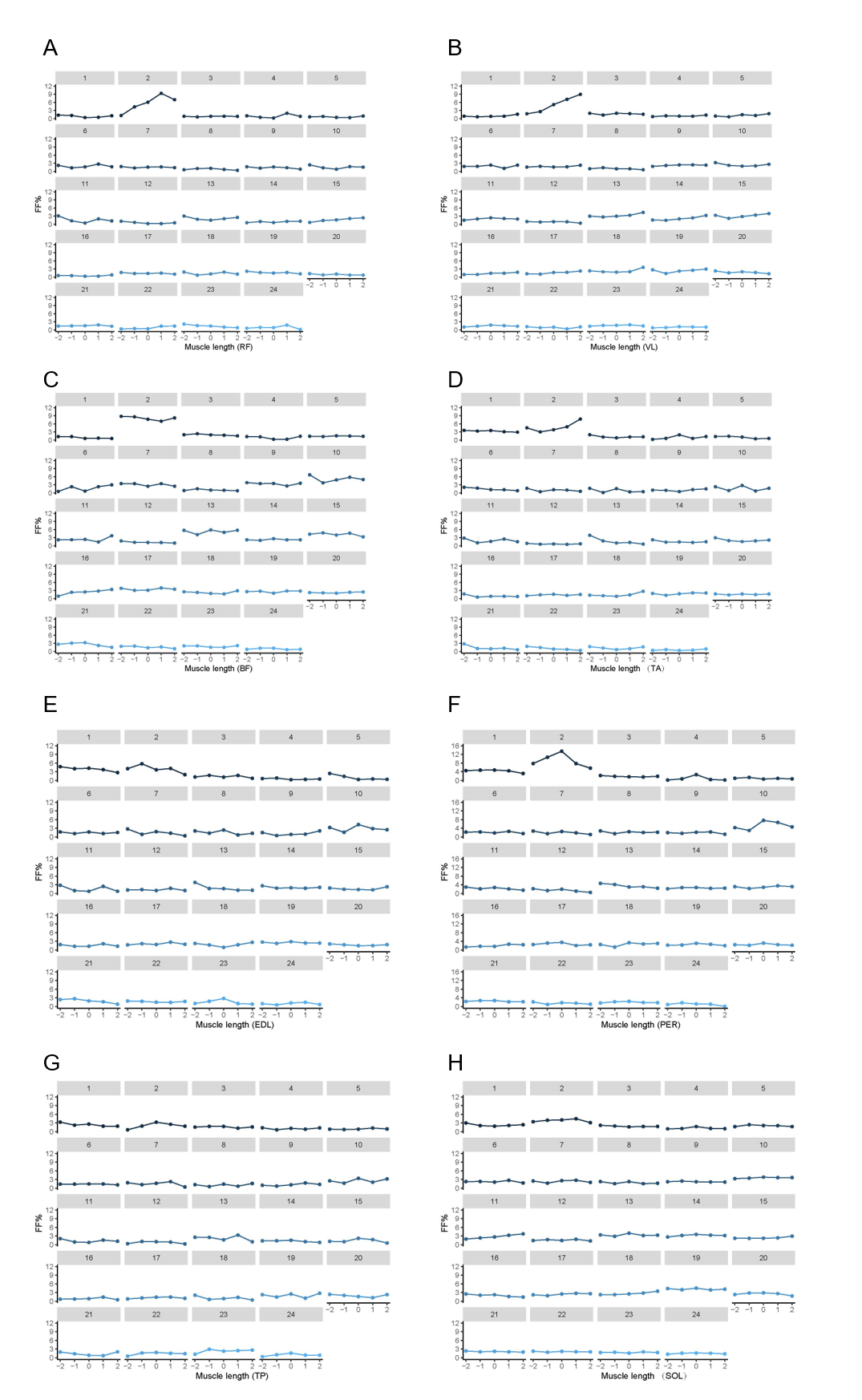


RF, rectus femoris; VL, vastus lateralis; BF, biceps femoris long head; TA, tibialis anterior; EDL, extensor digitorum longus; PER, peroneal group; TP, tibialis posterior; SOL, sole

Figure S**5** Population-average modeling for MRI FF for leg muscles.

Progression of muscle fat replacement with increasing ages in DMD patients using 3-knot b-splines reveals common inflection points and distinct progression patterns for leg muscles (A). (B-C) Age-associated changes in FF of the TA muscle, with population-level progression patterns estimated through NCDF model (B) and PL model (C). Filled circles denote FF values obtained from individual participants. Solid red and green lines show the PL model and NCDF model fit, respectively. FF, fat fraction; TA, tibialis anterior; NCDF, normal cumulative distribution function; PL, piecewise linear.
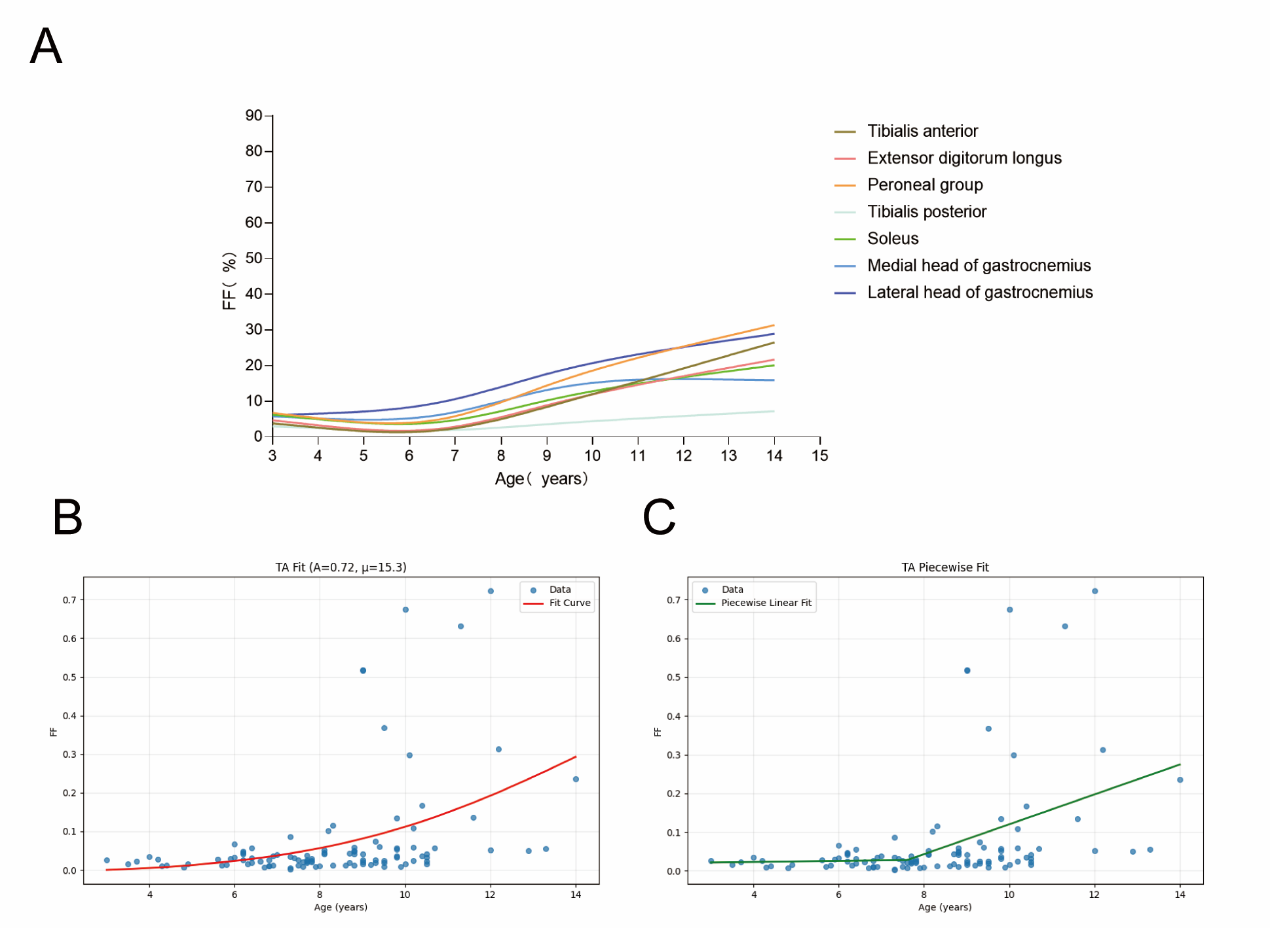

Supplement: Supplementary file 1 — Table S1: Slice selections for measuring specific skeletal muscles. Table S2: Comparison of muscle MRI FF and histopathological fatty infiltration percentage. Table S3: Reproducibility of MRI measurement in three observers. Table S4: Consistency of MRI measurement between three observers. Table S5: Correlation between fat fraction of MRI and ambulatory function. Table S6: Differences in FF values of thigh and calf muscles along the length axis. Table S7: Analysis of inhomogeneity of fat distribution over the length of thigh muscles. Table S8: The comparison between PL model and NCDF model in DMD and BMD group. Figure S1: Overview of participant enrolment and study design. Figure S2: Four selected slices for ROI placement of different muscle compartments. Figure S3: Fat distribution profiles along the length of muscles in DMD patients. Figure S4: Profiles of fat distribution along the length of controls. Figure S5: Population‐average modelling for MRI FF for leg muscles. [file JCSM-17-e70205-s001.docx]
